# Decreased plasma Chromogranin A361-372 (Catestatin) but not Chromogranin A17-38 (Vasostatin) in female dogs with bacterial uterine infection (pyometra)

**DOI:** 10.1186/s12917-015-0328-6

**Published:** 2015-01-31

**Authors:** Supranee Jitpean, Mats Stridsberg, Ann Pettersson, Odd V Höglund, Bodil Ström Holst, Ragnvi Hagman

**Affiliations:** Department of Clinical Sciences, Swedish University of Agricultural Sciences, SE-750 07 Uppsala, Sweden; Department of Surgery and Theriogenology, Faculty of Veterinary Medicine, Khon Kaen University, Khon Kaen, 40002 Thailand; Department of Medical Sciences, Uppsala University, SE-751 85 Uppsala, Sweden

**Keywords:** Pyometra, Chromogranin A, Catestatin, Vasostatin, C-reactive protein, Biomarker, Dog, Uterine infection

## Abstract

**Background:**

Pyometra often induces systemic inflammatory response syndrome (SIRS) and early diagnosis is crucial for survival. Chromogranin A (CgA) is a neuroendocrine secretory protein that is co-released with catecholamines from the adrenal medulla and sympathetic nerve endings. A prognostic value of CgA has been found in humans that are critically ill or that have SIRS associated with infection. CgA has not yet been studied in dogs with bacterial infection. The aim of the study was to investigate CgA, measured by Chromogranin A361-372 (Catestatin; Cst) and Chromogranin A17-38 (Vasostatin; VS) in healthy dogs and in dogs with pyometra.

**Results:**

Fifty dogs with pyometra, sampled prior to surgery and 64 healthy female dogs were included. In 19 pyometra cases, blood samples were also collected postoperatively. Concentrations of Cst and VS were measured in heparinised plasma and Cst also measured in EDTA plasma, by in-house radioimmunoassays. Student’s t-test and Wilcoxon two-sample test was used to test for differences between dog groups. Pre- and postoperative samples in dogs with pyometra were analysed by paired t-test. Pearson correlation was used to investigate associations of laboratory variables and hospitalization. *P* < 0.05 was considered significant.

Concentrations of Cst were decreased in pyometra dogs (mean ± SE, 1.01 ± 0.05 nmol/L) compared to healthy dogs (mean ± SE, 1.70 ± 0.03 nmol/L) (p ≤ 0.0001). VS concentrations did not differ significantly between dogs with pyometra (0.40 ± 0.04 nmol/L) and healthy dogs (0.42 ± 0.03 nmol/L). Mean ± SE pre- and postoperative concentration of Cst (1.0 ± 0.04 nmol/L and 0.9 ± 0.2 nmol/L) and VS (0.36 ± 0.04 nmol/L and 0.36 ± 0.04 nmol/L) in dogs with pyometra did not differ significantly. Neither Cst nor VS concentrations were associated with duration of hospitalization and were not significantly different in the four dogs with pyometra that had prolonged (≥3 d) postoperative hospitalization.

**Conclusion:**

Concentrations of Cst, but not VS, were decreased in pyometra. Cst and VS concentrations before and after ovariohysterectomy did not differ significantly and were not associated with duration of hospitalization. Further studies are warranted to evaluate a possible diagnostic or prognostic value for Cst and VS.

**Electronic supplementary material:**

The online version of this article (doi:10.1186/s12917-015-0328-6) contains supplementary material, which is available to authorized users.

## Background

One of the most common diseases of intact female dogs is bacterial uterine infection and inflammation leading to pus accumulating in the uterus (pyometra). The disease induces systemic inflammatory response syndrome (SIRS) *i.e.* sepsis in most cases with organ dysfunctions and death as possible consequences [1]. Early detection and proper treatment is therefore crucial for survival. The overall incidence of pyometra is 19% in female dogs up to 10 years of age, but despite often leading to sepsis, the mortality rate (3-4%) is relatively low [2-4].

In human medicine, acute phase proteins (APPs) such as, C-reactive protein (CRP) and Procalcitonin have been used as biomarkers for diagnostic and prognostic purposes in various diseases [5-9]. Similarly, in veterinary medicine, biomarkers and APPs are gaining interest in research studies [10-16]. One of the major APPs in dogs, CRP, has been shown to increase in dogs with pyometra [16].

Chromogranin A (CgA) is a neuroendocrine secretory acidic and water soluble protein, which belongs to the granin family [17]. It is a prohormone of several functional peptides such as Catestatin and Vasostatin and is coreleased with catecholamines from the adrenal medulla and sympathetic nerve endings [18,19]. Granins contain multiple protease and peptidase cleavage sites and are named based on their biological activities, e.g. Catestatin, Vasostatin and Pancreastatin [20-23]. Catestatin (Cst) and Vasostatin (VS), derived from CgA are known to act as cardio-protectants in case of ischaemic/reperfusion injury [24,25]. Several studies have shown that concentrations of stress-related neurochemicals, e.g. catecholamines that are released together with CgA, increase in sepsis [26-29].

Measurement of CgA is useful for diagnostic and predictive purposes in various syndromes and diseases in humans such as sepsis, neuroendocrine tumors and heart disease [30-34]. Moreover, increased CgA concentration has prognostic value in human patients suffering from stress, critical illness or infection associated with SIRS [30-32]. So far, concentrations of CgA have not been investigated in dogs with infection or before and after surgery. Because measurements of intact CgA are not possible yet in dogs, measurements of Cst and VS concentrations using in-house methods that have been validated for use in this species were applied [35].

The aims of the present study were to investigate concentrations of CgA, measured by Cst and VS, in healthy dogs and in dogs with pyometra, and before and after surgery to evaluate a possible diagnostic or prognostic value. CRP, hospitalization and laboratory variables were also investigated to evaluate whether they were associated with either Cst or VS.

## Methods

### Ethical approval

The study was approved by the Uppsala Local Ethical Board (permission number C413/12), and a written informed owner consent was obtained before inclusion in the study.

### Animals

In total, 114 female dogs were included in the study. Of these, 50 were bitches with pyometra (23 breeds) and 64 were healthy dogs (22 breeds) (for more details on the included breeds see Additional file 1). A complete physical examination was performed by the veterinarian in charge, and the results recorded in a special form and in the patient records. The preliminary diagnosis of pyometra was based on case history data, findings on physical examination, laboratory test results, and diagnostic imaging by either abdominal ultrasonography or radiology or both. All bitches with pyometra were treated by ovariohysterectomy (OHE) at the University Animal Hospital (UDS), Swedish University of Agricultural Sciences (SLU), Uppsala, during the study period 2009–2013.

The diagnosis pyometra was confirmed by postoperative macroscopic identification of a pus-filled uterus together with histopathological diagnosis and/or positive bacterial culture from the uterine content. Histopathological examination of formaldehyde-fixed uteri and ovaries was performed at the Department of Biomedical Sciences and Veterinary Public Health, SLU, Uppsala. Samples for bacterial identification and drug sensitivity were immediately collected from uterine content by using sterile fibre cotton swabs (Culturette; Becton-Dickinson AG, Stockholm, Sweden). The methods were performed at the accredited laboratory, Section of Bacteriology, National Veterinary Institute (SVA), Uppsala, Sweden as earlier described [36]. Dogs diagnosed with Mucometra, Hydrometra, Cystic endometrial hyperplasia (CEH) or uterine tumours were excluded. Prolonged postoperative hospitalization was defined as ≥ 3 days, as described in our previous study (regular postoperative hospitalization after OHE at UDS is 1–2 days) [16].

### Blood sampling and laboratory tests

#### Hematological and biochemical analysis

Blood was obtained from dogs with pyometra immediately or up to two hours before surgery and from the healthy control dogs. From 19 of the 50 dogs with pyometra, blood samples were collected both prior to and 24 ± 3 h after surgery. The samples were aseptically collected from the distal cephalic vein into heparinised, EDTA and nonadditive collection tubes (Vacutainer^®^, Becton-Dickinson, Stockholm, Sweden). Hematological analyses (Total white blood cell count (WBC), including differential counts, Hematocrit (PCV) and Hemoglobin (Hb) were performed (Advia 2120; Siemens Healthcare Diagnostics, Deerfield, IL, USA). After centrifugation and separation of serum and plasma, biochemical and CRP analyses- Albumin, Bile acids, Alanine aminotransferase (ALT), Glucose, Blood urea nitrogen (BUN), and Creatinine- were performed (Abbott Archtect c4000, Abbott Park, IL, USA). All analyses were conducted according to routine methods at the Clinical Pathology Laboratory, UDS, SLU, Uppsala, Sweden. Unused serum and plasma was transferred in aliquots of 200 μL into cryogenic vials (NuncCryoTubes, VWR International, Stockholm, Sweden), and freeze-stored at −80°C until analysis of CgA.

#### Biomarker analyses

CRP was analysed by an automated assay (High Linearity CRP, Randox Laboratories, Crumlin, United Kingdom) performed on Abbot Architect (Abbott Architect c4000, Abbott Park, IL, USA). The method has previously been validated for dogs [37,38]. The limit of quantification was 5 mg/L with a mean intra- and interassay variation of 1.4% and 2.4%, respectively. Samples with concentrations of CRP- above 217 and 225 mg/L for the two lots used- were autodiluted 1:3 with 0.9% NaCl and reanalysed to obtain exact values.

Cst and VS were measured in heparinised plasma by radioimmunoassays specific for Cts and VS [39,40] and previous validated for use in dogs [35]. None of the commercial methods to determine CgA can be used for measurements in dogs (and other animals), due to the large differences in amino acid sequences between different species. However, defined parts of the CgA molecule have higher amino acid homology and methods that are specific to these parts can be used to measure CgA in several animal species, including dogs [35,39]. This cross-reactivity was first shown in a study using the same VS assay as in our study for measurements of CgA in the human, bovine, equine, porcine and ovine species [39]. Other parts of the CgA molecule have been investigated for potential cross reactivity and it was found that both VS and Cst could also be used in dogs [35]. These assays are described in detail in a previous study and are in-house assays performed at the Research Department of Clinical Chemistry, Uppsala University Hospital, Sweden [40]. Furthermore, it has been shown that there is a positive correlation between VS and intact CgA, which justifies the use of VS to estimate CgA concentrations [39,40]. For Catestatin, there is a cross reactivity that allows us to use also this part of the CgA molecule to estimate of CgA concentrations [35].

The validation study of CgA in dogs and cats was performed by using extracts of CgA from the adrenal gland since it contains high concentrations of CgA [35]. The coefficient of variation (CV) was 3.6% for Cst and 8.8% for VS. To test preanalytical conditions, analysis of Cst was performed in both EDTA and heparinised plasma from 45 dogs with pyometra.

### Statistical analyses

Statistical analyses were performed by using Minitab software programs for Windows version 16 (Minitab Inc., State College, PA, USA). The Anderson-Darling test was used to evaluate normality of data distribution. For normally distributed data, Student’s t-test was used to test differences of hematology, biochemistry, Cst and VS variable between dogs with pyometra and healthy dogs. Concentrations of Cst and VS in pre- and postoperative samples in the pyometra group were compared by using paired t-tests. In the healthy dogs that had concentrations of CRP lower than 5 mg/L, the concentration was set to half the limit of quantification (2.5 mg/L) for the statistical analyses. A Wilcoxon two-sample test was used for analysis when most healthy dogs had CRP concentrations below the lower measurable concentration. In the dogs with pyometra, Pearson’s correlation was used to evaluate associations between Cst and VS and other variables - including age, weight, hematology, biochemistry, CRP, and duration of hospitalization. Pearson’s correlation was used to investigate associations between Cst and VS in heparinised and EDTA plasma. The significance level was set at p < 0.05 for all tests used.

## Results

### Dogs

The mean age (± SD) was significantly higher in the pyometra group (7.2 ± 2.5 years, n = 49) compared to the control group (5.4 ± 3.5 years, n = 54, p = 0.003). The mean weight (± SD) was significantly higher in the dogs with pyometra (27.5 ± 9.5 kg, n = 50) compared to the healthy dogs (21.6 ± 9.4, n = 64, p = 0.001). Neither Cst nor VS concentrations were correlated to age or weight in the healthy dogs.

### Laboratory variables

In the pyometra group, WBC, Neutrophils, Band neutrophils and Monocyte were increased compared to the control group. Meanwhile, Hb, PCV, Lymphocytes and Albumin were significantly decreased in dogs with pyometra compared to healthy group (Table 1). Most dogs in the pyometra group had Creatinine and BUN concentrations within the reference range for healthy dogs (Table 1).

**Table 1:** **Hematology and biochemistry variables in 50 dogs with pyometra (pyometra group) and 64 healthy dogs (control group)**

**Variable**	**Pyometra group**	**Control group**	**p value**	**Reference range** ^**†**^
	**Mean ± SE (n) (range)**	**Mean ± SE (n) (range)**	**(student’s t-test)**	
Hemoglobin (g/L)	131 ± 4 (47)	159 ± 3 (48)	<0.0001	132 - 199
	(89 - 192)	(111 - 203)		
Hematocrit (PCV) (%)	37 ± 1 (47)	45 ± 0 (48)	<0.0001	38 - 57
	(25 - 53)	(32 - 59)		
WBC (x10^9^/L)	19.7 ± 2.2 (48)	9.3 ± 0.3 (56)	<0.0001	5.8 - 16.0
	(4.9 - 106.4)	(4.5 - 16.6)		
Neutrophils (x10^9^/L)	11.9 ± 1.8 (46)	5.5 ± 0.2 (56)	0.001	3.0 - 11.5
	(0.1 - 85.1)	(1.1 - 10.7)		
Band neutrophils (x10^9^/L)	4.0 ± 0.8 (44)	0.4 ± 0.2 (42)	<0.0001	0 - 0.3
	(0 - 22.4)	(0 - 9.0)		
Lymphocytes (x10^9^/L)	1.7 ± 0.1 (46)	2.3 ± 0.1 (56)	0.005	1.4 - 4.8
	(0 - 4.4)	(0.5 - 5.1)		
Monocytes (x10^9^/L)	1.8 ± 0.2 (46)	0.7 ± 0.2 (56)	<0.0001	0.2 - 1.4
	(0.2 - 5.4)	(0 - 9.2)		
Eosinophils (x10^9^/L)	0.4 ± 0.1 (45)	0.8 ± 0.1 (56)	0.02	0.1 - 1.2
	(0 - 3.6)	(0 - 4.4)		
Basophils (x10^9^/L)	0 ± 0 (45)	0.05 ± 0.02 (56)	0.06	0 - 0.1
	(0 - 0.3)	(0 - 1.0)		
Albumin (g/L)	26.2 ± 0.7 (43)	30.0 ± 0.4 (46)	<0.0001	29 - 39
	(18 - 35)	(22 - 34)		
Bile acids (μmol/L)	3.4 ± 0.6 (42)	9.9 ± 2.5 (41)	0.01	0 - 12
	(0.3 - 17.3)	(0 - 63.5)		
ALT (μkat/L)	0.5 ± 0.09 (45)	0.6 ± 0.03 (52)	0.66	0 - 1.3
	(0 - 3.9)	(0.1 - 1.3)		
Glucose (mmol/L)	5.0 ± 0.18 (42)	5.1 ± 0.1 (41)	0.4	4.5 - 5.8
	(1.5 - 7.3)	(3.7 - 6.8)		
BUN (mmol/L)	3.7 ± 0.2 (43)	5.5 ± 0.3 (41)	<0.0001	2.5 - 8.8
	(1.5 - 7.5)	(3.0 - 14)		
Creatinine (μmol/L)	65.7 ± 3.1 (43)	72 ± 2 (43)	0.2	40 - 130
	(43 - 155)	(50 - 96)		

WBC = Total white blood cell count, ALT = Alanine aminotransferase, BUN = Blood urea nitrogen.

^†^Reference range (at the Clinical Pathology Laboratory, University Animal Hospital, Swedish University of Agricultural Sciences, Uppsala).

### Biomarkers

Concentrations of Cst were significantly decreased in dogs with pyometra compared to healthy dogs (Table 2). VS concentrations did not differ between the two groups of dogs (Table 2). CRP concentrations were significantly higher in the pyometra group compared to the control group (Table 2). Concentrations of Cst and VS were moderately positively correlated (r_p_ = 0.3, p = 0.03).

**Table 2:** **Concentrations of Catestatin (Cst) and Vasostatin (VS), and C-reactive protein (CRP) in 50 dogs with pyometra (Pyometra group) and 64 healthy female dogs (control group)**

**Variable**	**Pyometra group**	**Control group**	**p value**
	**Mean ± SE (n) (range)**	**Mean ± SE (n) (range)**	
Cst	1.0 ± 0.05 (50)	1.7 ± 0.03 (64)	<0.0001^*^
(nmol/L)	(0.10 - 1.66)	(1.07 - 2.3)	
VS	0.40 ± 0.04 (48)	0.42 ± 0.30 (64)	0.7
(nmol/L)	(0.14 - 1.34)	(0.10 - 1.34)	
CRP	174.9 ± 20.7 (35)	<4.5 ± 1.4 (21)	0.001^¶^
(mg/L)	(2.5 - 370)	(2.5 - 28)	

^*^p value = Student’s t-test, ^¶^p value = Wilcoxon two sample test.

Cst concentrations were moderately positively correlated with Hb and PCV in the pyometra group (r_p_ = 0.3, p = 0.03 and r_p_ = 0.3, p = 0.03, respectively).

CRP concentrations were not correlated with Cst or VS in the pyometra group.

Cst and VS concentrations in dogs with pyometra before and 24 ± 3 h after surgery did not differ significantly (Table 3). Cst concentrations prior to surgery in the dogs with pyometra with prolonged postoperative hospitalization (n = 4) did not differ significantly from those with regular postoperative hospitalization (≤ 2 days, n = 15, mean ± SE, 0.91 ± 0.07 nmol/L and 0.82 ± 0.24 nmol/L, respectively). VS concentrations before surgery did not differ significantly between bitches with prolonged hospitalization and bitches with regular postoperative hospitalization (mean ± SE concentration, 0. 57 ± 0.14 nmol/L and 0.32 ± 0.03 nmol/L, respectively).

**Table 3:** **Concentrations of of Catestatin (Cst) and Vasostatin (VS) in 19 dogs with pyometra sampled before surgery (preoperative) and after surgery (postoperative)**

**Variable**	**Preoperative**	**Postoperative**	**p value**
	**Mean ± SE (n) (range)**	**Mean ± SE (n) (range)**	**(Paired t-test)**
Cst	0.89 ± 0.07 (19)	0.86 ± 0.04 (19)	0.6
(nmol/L)	(0.10 - 1.28)	(0.61 - 1.21)	
VS	0.36 ± 0.04 (18)	0.36 ± 0.04 (18)	0.9
(nmol/L)	(0.16 - 0.95)	(0.1 - 0.73)	

Cst and VS concentrations in heparinised plasma and EDTA plasma were strongly positively correlated (r_p_ = 0.825, p < 0.001).

## Discussion

Concentrations of plasma Cst were decreased in dogs with pyometra. This result was somewhat unexpected, as it is not in line with increased CgA blood concentrations found in critically ill and intensive care human patients with severe sepsis and other diseases [30-32,41]. Most studies in humans include patients investigated during early stages of disease, but in pyometra, the disease may have developed during several days or weeks before admission to an animal hospital, which could explain the decreased levels of Cst in dogs with pyometra.

Another explanation for the lower concentrations found in dogs with pyometra could be that negative feedback control of Cst leads to inhibited secretion of catecholamines and CgA, and subsequently decreased CgA concentrations. Several studies in humans have reported that Cst is a potent and specific cholinergic antagonist in chromaffin cells, and increased Cst concentration could thereby inhibit catecholamine secretion [23,42]. Such negative feedback would be more likely to occur in individuals with chronic (pyometra) as opposed to acute disease (severe illness or sepsis in the studied human patients).

Inflammation and sepsis may lead to impaired coagulation [43] and has been shown to occur in bitches suffering from pyometra [44]. Disturbed coagulation has previously been reported as one of many pathologies in dogs with the disease [44]. This may be because the inflammatory response induced in pyometra is multifaceted. A study on gene activation in the uterine tissue in dogs with pyometra found over 800 upregulated genes including genes encoding proteinases, proteolyses, disintegrin and metalloproteinase with thrombospondin type 1-like motifs (ADAMTS) families and thrombospondin-4 regulating gene [45]. In a study in humans, thrombin caused decreased CgA concentrations in the circulation and it was also shown that several proteinases were involved in this process [46]. These findings indicate that thrombin could play a role in causing decreased Cst concentrations. However, mechanisms and interrelationship of CgA, thrombin and severe illness have not yet been studied in detail in dogs.

Possibly there is a system for downregulation of CgA in response to infection, at least in some animal species. Because this is the first report of CgA in a disease related to sepsis in dogs, the diagnostic and predictive value of CgA needs to be further studied for such evaluation to be possible. In critically ill human patients, serum CgA concentrations were correlated with concentrations of serum creatinine [32]. In the present study, no such correlation could be found, possibly because most diseased dogs studied here had creatinine concentrations within the normal reference range for healthy dogs. Cst and VS concentrations were correlated, albeit not strongly but VS concentrations did not differ significantly between the pyometra group and the control group. This finding is in contrast to results in humans where increased VS blood concentrations were related to sepsis and survival [47,48]. Differences in results among studies may be due to interspecies variation or because the dogs included in our study suffered from less severe disease compared to the studied humans.

Why Cst concentrations but not VS concentrations were decreased remains to be determined. The relation between intact CgA and its fragments is complex and although this has not been shown, one possible explanation of the demonstrated differences found between Cst and VS could be that Cst measurement comprises both intact CgA and Cst as well as other possible fragments of CgA that contain the Cst part. Because the assay is expressed in molar units it measures the number of molecules present in the sample *i.e*. the assay most probably measures the total CgA content. The same is true for VS measurements with the currently used assay, but when comparing results from these two assays (Cst and VS), diverging results were found. As mentioned earlier, Cst and VS have different biological activities which could also partly explain this divergence [49-54]. The metabolic clearance rate of the two molecules may likewise differ, which in turn can influence the number of molecules that are present in the sample. There are thus several reasons that could explain the resulting two different CgA levels measured by the Cst and VS assays.

The concentration of Cst in healthy dogs, using the same method as used here, was lower in a study of female and male Beagle dogs [55]. It is possible that CgA concentrations vary by breed, which could explain why the results of the present study, including results from many breeds, differed from the study that included only dogs of one breed (Beagle). Further, in a study in humans, it was reported that gender influenced the concentration of CgA in plasma [56]. Our study included only female dogs while the previous study included both genders and this may also have contributed to the differences in CgA concentrations between the present and the earlier report in dogs [55].

Measuring concentrations of the entire CgA molecule in dogs would give more accurate information of CgA compared to measuring Cst and VS, but currently such a method is not available. For more knowledge and understanding of mechanisms and roles of CgA in dogs with infection, further studies are necessary.

That CRP concentrations were increased in pyometra is in accordance with findings of several other studies, and is a consequence of the severe inflammatory response induced [15,16,57]. CRP was, however, not correlated with CgA (Cst or VS) and the CgA concentrations measured before surgery did not differ from concentrations measured after surgery in spite of a possible response to surgery. Additionally, the length of hospitalization was not associated with the concentrations of Cst or VS. These results are in contrast to a study in humans with sepsis which found that more severe systemic inflammation, indicated by higher CRP, was linked with higher concentrations of CgA and that survivors had a lower CgA concentrations compared to nonsurvivors [32]. The reason for the lack of association between CgA and CRP or postoperative hospitalization (as a measurement indicator of morbidity) is unknown, and an association between CgA and mortality remains to be investigated because no dogs died of the pyometra in current study.

In pyometra, the PCV, Hb, Lymphocyte and Albumin concentrations were decreased whereas WBC, Neutrophils, Band neutrophils, and Monocytes were increased in dogs with pyometra compared to healthy dogs. These results illustrate the systemic illness induced by the disease, and shows that the dogs studied were indeed severely affected [4].

CgA in heparinised plasma was strongly positively correlated with CgA in EDTA plasma. These results show that both heparinised and EDTA plasma can be used for Cst analysis in dogs.

A limitation of the study was that some the samples had been freeze-stored up to 5 years before analysis of Cst and VS. This duration however is unlikely to have affected the results as samples from both groups were collected during the same time period and CgA is stable at room temperature and minimally affected by repeated thawing-refreezing cycles [58]. CRP is also considered stable in room temperature and when freeze stored [59]. That age and weight differences between the two study groups could have somewhat influenced the results cannot be completely excluded, but because the age and weight were not correlated with CgA, as tested in the 64 healthy bitches, a notable effect on the results presented here is not expected.

It has been shown that Cst is upregulated in skin during bacterial infection [60], but whether local infection of the uterus has any effect on Cst has not yet been investigated. Based on the present results, it could be speculated that CgA is more likely to be released in response to systemic inflammation as it did not decrease after surgical removal of the infected uterine tissue. However, Cst might still be clinically valuable if used together with other markers for diagnostic and prognostic purposes in dogs with severe illness induced by pyometra. The value of CgA and its products when measured in dogs with sepsis remains to be determined.

## Conclusion

CgA concentrations were decreased in pyometra, as measured by Cst. Concentrations of VS and Cst were correlated but VS concentrations did not differ between the diseased and healthy groups of dogs studied. None of studied variables had predictive value for prognosis as measured by correlation with postoperative hospitalization or when compared between dogs with prolonged and normal hospitalization. Further studies are needed to investigate whether Cst or VS derived of CgA are related to severity or mortality.

## Additional file

Additional file 1:
**Dog breeds in the Pyometra and Control group.** Of 114 bitches enrolled in the study, 50 were bitches with pyometra (23 breeds) and 64 were healthy bitches (22 breeds).
