# Supplementary material for: Decreased plasma Chromogranin A361-372 (Catestatin) but not Chromogranin A17-38 (Vasostatin) in female dogs with bacterial uterine infection (pyometra)
Source: BMC Vet Res. 2015 Jan 31;11:14. doi: 10.1186/s12917-015-0328-6 (PMC4318355; doi:10.1186/s12917-015-0328-6)
Supplement: Additional file 1: — Dog breeds in the Pyometra and Control group. Of 114 bitches enrolled in the study, 50 were bitches with pyometra (23 breeds) and 64 were healthy bitches (22 breeds). [file 12917_2015_328_MOESM1_ESM.docx]

**Additional file 1**

| **Breed** | **Pyometra group (n)** | **Control group (n)** |
| --- | --- | --- |
| Airedale Terrier | 1 | 1 |
| Beagle | 1 | 10 |
| Bedlington Terrier | 0 | 1 |
| Bernese Mountain Dog | 2 | 2 |
| Border Collie | 0 | 2 |
| Boston Terrier | 1 | 0 |
| Boxer | 1 | 0 |
| Ca de Bestiar | 1 | 0 |
| Cane Corso | 1 | 0 |
| Cavalier King Charles Spaniel | 3 | 0 |
| Chow Chow | 1 | 0 |
| Collie | 3 | 0 |
| Dachshund | 0 | 2 |
| English Springer Spaniel | 2 | 3 |
| Flat Coated Retriever | 2 | 6 |
| German Pointer | 0 | 1 |
| German Shepherd Dog | 4 | 3 |
| Golden Retriever | 13 | 4 |
| Hovawart | 1 | 1 |
| Irish Setter | 1 | 0 |
| Jack Russel Terrier | 0 | 1 |
| Labrador Retriever | 0 | 12 |
| Mixed breed | 3 | 6 |
| Munsterlander | 1 | 0 |
| Norfolk Terrier | 0 | 1 |
| Nova Scotia Duck Tolling Retriever | 0 | 1 |
| Rottweiler | 2 | 2 |
| Schnauzer | 2 | 0 |
| Staffordshire Bull Terrier | 2 | 2 |
| Standard Poodle | 1 | 1 |
| Swedish hound | 1 | 0 |
| Toy Poodle | 0 | 1 |
| Welsh Springer Spaniel | 0 | 1 |
